# Federating AI-related regulations for human therapeutics: an AI-enabled, continuously updating regulatory intelligence system

**DOI:** 10.3389/fdsfr.2026.1718379

**Published:** 2026-03-09

**Authors:** Rominder Singh, Mukesh Pareek, Michael Prokle, Eduardo Sanchez, Uwe Hohgrawe, Jared R. Auclair

**Affiliations:** 1 College of Professional Studies, Northeastern University, Boston, MA, United States; 2 LunarTree, Dover, DE, United States

**Keywords:** artificial intelligence (AI), drug regulation, federated access, framework, human therapeutics, regulatory intelligence

## Abstract

Global regulation of artificial intelligence (AI) in healthcare remains highly fragmented. Over 1,000 national AI-related regulations and policy frameworks have been introduced across more than 70 countries, most originating from high-income nations. While major drug regulatory authorities, including those in the United States and European Union, and others like WHO, have issued guidance on AI-enabled systems, platforms, and products, no unified source exists that compiles or compares how these bodies govern the broader AI-enabled therapeutic ecosystem. This lack of alignment contributes to disparities in interpretation and implementation, potentially delaying global access to novel AI-based therapies. To address this gap, we developed AI-enabled, continuously updated regulatory intelligence system (AICURIS), a comprehensive AI-enabled regulatory intelligence system trained initially on AI-related regulations from sentinel regulatory authorities (e.g., FDA, EMA, WHO), designed to continuously monitor and classify AI-relevant regulatory content and to enable structured comparison, identification of alignment and divergence, and evidence-informed discussion on regulatory convergence as its coverage expands across jurisdictions. Using over 400,000 regulatory documents published since 2019, an AI-enabled hybrid semantic similarity and keyword scoring model achieved 95% recall for high-confidence AI-related content. The following results summarize the key findings from the analysis of AI-related regulatory documents across three major agencies (United States Food and Drug Administration (FDA), European Medicines Agency (EMA), and the World Health Organization (WHO)). The findings are presented in six parts: (1) document corpus composition and filtering outcomes, (2) model optimization, (3) classification model performance, (4) classification ensemble model validation, (5) cross-agency bias mitigation, and (6) real-time monitoring capabilities. This study demonstrates that AI-powered, data-driven approaches can effectively federate AI-related drug regulations across jurisdictions, reducing global disparities, and enabling more equitable, collaborative, and efficient human therapeutics innovation.

## Background

Artificial intelligence (AI) and machine learning (ML) are reshaping the pharmaceutical and life sciences industries, powering a connected ecosystem across a product’s life cycle, spanning early drug discovery, clinical trial optimization, and post-market surveillance. This transformation holds the potential to accelerate innovation, reduce development costs, improve patient safety, and advance access to personalized and equitable therapies worldwide. However, this rapidly evolving field faces critical questions about optimal regulation. AI-related regulations, guidelines, and policy initiatives span diverse topics, including ethics, risk management, data governance, and AI system accountability (Johnson, 2025). These regulations are highly fragmented across countries and health authorities, raising concerns about regulatory consistency and effectiveness. For instance, the United States alone exhibits considerable regulatory overlap and fragmentation, with the FDA releasing over 20 AI-related policy documents by mid-2025, alongside complementary but distinct frameworks from the National Institute of Standards and Technology (NIST) and the Department of Defense (DoD), and additional complexity from state-level efforts such as California’s numerous AI-related legislative bills (Gottlieb, 2024; Johnson, 2025; Loper Bright Enterprises v. Raimondo, 2024; USAGov, 2025).

The current broad yet disjointed policy environment presents significant risks for developing and deploying regulations tailored to AI-enabled human therapeutics. The main concern lies not in the number of evolving regulations but in the potential inconsistencies, duplications, and ambiguities across sectors and jurisdictions. Regulatory agencies publish guidance documents, discussion papers, and policy frameworks in disparate formats and locations, creating a fragmented information landscape. This regulatory dissonance could hamper innovation, increase development costs, delay regulatory approvals, and discourage investment (Warraich et al., 2025). This may particularly impact startups, small companies, and low- and middle-income countries (LMICs) that lack resources for developing regulations related to AI in human therapeutics.

Addressing this challenge necessitates comprehensive understanding of the fragmented global regulatory landscape (Singh et al., 2025). Existing regulatory intelligence efforts predominantly rely on predefined, point-in-time analyses of AI policies within specific jurisdictions or therapeutic domains, employing manual document processing with fragmented coverage, thus limiting timeliness and comprehensiveness of available regulatory insights (Tsopra et al., 2021; U.S. Food and Drug Administration, 2024a; U.S. Food and Drug Administration, 2024b).

The first comprehensive, AI-enabled continuously updated regulatory intelligence system.

This work introduces a regulatory intelligence system addressing fragmentation through three innovations: (1) automated pipelines continuously monitoring FDA, EMA, and WHO sources in near real-time, (2) semantic similarity and keyword-based classification detecting AI-relevant content across diverse regulatory terminologies, and (3) an extensible architecture supporting adaptation to new regulatory bodies and emerging themes without complete redesign.

The system is intended to inform human-led regulatory interpretation and dialogue, rather than to generate normative judgments or automated regulatory decisions, and its scope is expected to expand progressively to additional jurisdictions. The resulting comprehensive database enables three key stakeholder benefits: (1) policymakers can benchmark regulatory approaches across jurisdictions and identify harmonization opportunities; (2) regulators can monitor global AI regulatory trends and streamline reviews to reduce duplication and fill coverage gaps; (3) and various other stakeholders such as drug developers and biopharma companies can track evolving compliance requirements across multiple markets simultaneously, reducing regulatory uncertainty and compliance costs for AI-enabled drug development.

## Methodology

### Data acquisition and scope

**FIGURE 1:**
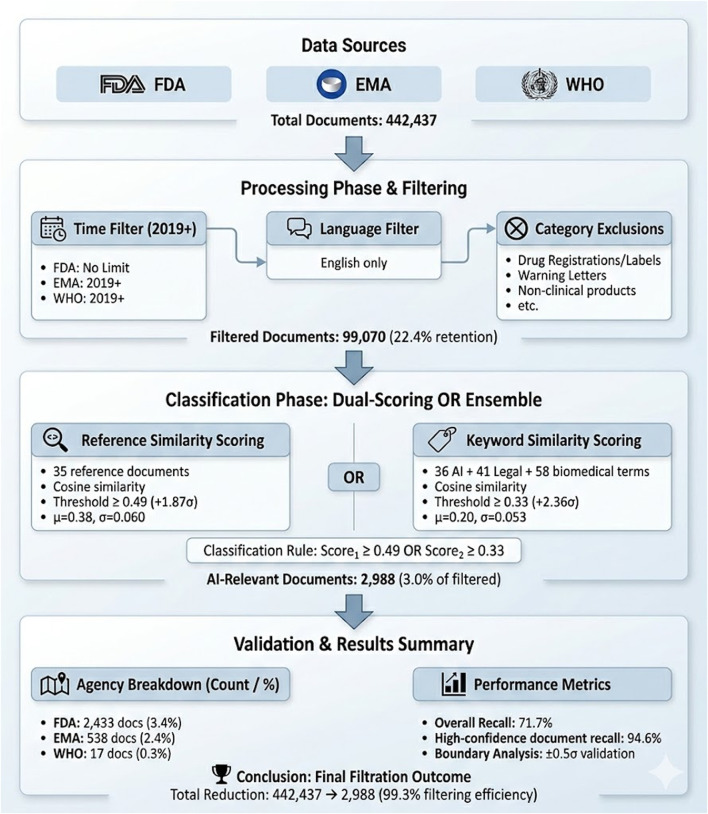
Data Funnel & End-to-End Methodology Overview from Data Collection to Validation & Results.

A scalable, automated data pipeline (Figure 1: Data Funnel & Methodology Overview) was developed to enable continuous monitoring and analysis of publicly available regulatory documents from the websites of the three major regulatory bodies, the FDA, the EMA, and the WHO. All data collection strictly adhered to each website’s terms of use and robots exclusion protocol (i.e., robots. txt directives). No proprietary, confidential, or access-restricted content was accessed or analyzed. These agencies were chosen based on their global regulatory influence, historical output, and increasing engagement with AI-relevant topics. Collectively, they regulate markets responsible for 83 percent of new pharmaceutical product launches from 2018 to 2023 (European Federation of Pharmaceutical Industries and Associations [EFPIA, 2024).

Over a 6-year period (2019–2025), 442,437 documents were identified for analysis across multiple formats, including guidance documents, regulations, white papers, reflection papers, concept notes, and technical reports. A scalable, automated data pipeline was developed using Python-based web scraping and PDF extraction tools to continuously monitor regulatory documents from FDA, EMA, and WHO websites (Artifex Software, 2025; Richardson, 2025). Content was restricted to English-language documents published between 2019–2025, with category filters applied to remove content unrelated to AI regulation (such as routine drug labels, standard pharmacovigilance reports, and administrative notices). This filtering process yielded a final corpus of 99,070 documents. (See Supplementary Methods Section 1 for detailed technical implementation, including web-scraping protocols, cloud infrastructure, and content extraction methodologies).

## Reference and test document sets

### Definition of AI-relevant content

AI-relevant documents were defined as those that discuss artificial intelligence, machine learning, or data science technologies within regulatory contexts. This includes documents that govern AI use in drug development or describe its application in regulatory operations. Technical AI literature without regulatory implications was excluded, including methodological research papers, algorithm development studies, and technical implementation reports.

### Reference document set development

To identify AI-relevant content within the 99,070-document corpus, a reference document set of 34 regulatory documents was developed through systematic review of AI-related regulatory guidance from FDA, EMA, and WHO, selected to represent diverse document types and AI applications across agencies. Included foundational documents include FDA’s “Using Artificial Intelligence & Machine Learning in the Development of Drug & Biological Products” discussion paper (2023), EMA’s “AI workplan to guide use of AI in medicines regulation” (2023), and technical reports on AI applications in manufacturing quality control and pharmacovigilance (International Council for Harmonisation, 2025; U.S. Food and Drug Administration, 2021; European Medicines Agency, 2023).

Additionally, a synthetic reference document was constructed by combining comprehensive keywords from AI, legal/regulatory, and biomedical domains to serve as a generalized concept anchor for similarity scoring, reducing reliance on exact terminology and improving recall for documents that reference AI concepts using non-standard phrasing.

(See Supplementary Methods Section 2 for complete reference document selection methodology and Supplementary Data File 1 for the full reference document list. See Supplementary Methods Section 3 for synthetic document construction details and Supplementary Data File 4 for complete keyword taxonomy).

### Test document set development

An independent test set of 127 AI-relevant documents was created through keyword-based sampling of FDA documents, followed by two-stage manual review to classify documents as high-confidence (n = 38, e.g., FDA’s “Considerations for the Use of Artificial Intelligence To Support Regulatory Decision-Making”) or relevant (n = 89, e.g., digital health frameworks mentioning AI capabilities) (U.S. Food and Drug Administration, 2021). The test set was constructed independently from the classification framework to ensure unbiased validation.

The test set was constructed exclusively from FDA documents for validation efficiency, as FDA represented the largest document corpus (∼85,000 documents) and its website search functionality enabled systematic keyword-based sampling. Cross-agency generalizability was subsequently validated through bias mitigation analysis (see Results). Due to resource constraints, precision metrics could not be estimated as this would require manual review of the 97% of documents classified as non-relevant. The 3.0% positive classification rate (2,988 documents) represents a manageable manual review burden for regulatory intelligence applications.

(See Supplementary Methods Section 4 for complete test set development methodology including two-stage review process, and Supplementary Data Files 3, 6 for test document lists).

### Classification model development

A dual-scoring classification model was developed combining semantic similarity scoring with keyword-based analysis to address the challenge that regulatory AI content often uses varied terminology across agencies and document types. Vector embeddings were generated from document content and compared against 35 reference documents (34 actual + 1 synthetic), while keyword similarity was scored across AI, legal/regulatory, and biomedical terminology domains (OpenAI, 2025; Schopf et al., 2022). Seven scoring configurations were systematically evaluated, combining: (1) semantic similarity to reference documents (average and maximum scores), (2) similarity to the synthetic ideal document, and (3) keyword-based scoring across domain-specific terminologies. OR-ensemble logic was used to combine complementary detection mechanisms, where a document is classified as AI-relevant if it exceeds the threshold in any constituent method.

Classifier performance was evaluated using three metrics: Test Recall (proportion of known AI-relevant documents correctly identified in the 127-document test set), High Priority Recall (performance on the 38 high-confidence documents), and Positive Rate (proportion of full corpus classified as AI-relevant, determining manual review burden).

Threshold optimization was performed through a systematic grid search to identify configurations achieving ≥70% Test Recall while minimizing Positive Rate. The optimization prioritized recall over precision, as missing critical AI regulatory requirements could delay drug development or lead to non-compliance, whereas reviewing classified documents (∼3,000 at a 3% Positive Rate) remains manageable for regulatory intelligence teams (He and Cheng, 2021; He and Garcia, 2009; Saito and Rehmsmeier, 2015).

(See Supplementary Methods Sections 5–7 for detailed scoring methodology, all tested configurations, complete performance metric formulations, grid search procedures, and recall prioritization rationale. See Supplementary Data File 4 for complete keyword taxonomies).

### Ensemble model validation via boundary analysis

To validate ensemble performance on ambiguous classification cases, a boundary analysis was designed focusing on documents with scores near decision thresholds—regions where classification uncertainty is highest and ensemble benefits should be most apparent. Documents within narrow (±0.01) and wide (±0.5σ) boundaries of each threshold were analyzed to quantify ensemble recall improvements versus individual methods.

(See Supplementary Methods Section 8 for complete boundary analysis methodology including boundary region definitions, metrics formulations, and validation procedures).

### Technical implementation and reproducibility

Complete source code, technical documentation, and replication instructions are provided in the publicly accessible GitHub repository (Supplementary Data File 5). This live demonstration enables independent validation of system performance on newly published regulatory documents.

(See Supplementary Methods Section 9 for complete technical implementation details including technology stack, cloud infrastructure, data pipelines, and reproduction parameters).

## Results

The following results summarize the key findings from the analysis of AI-related regulatory documents across agencies. The findings are presented in six parts: (1) document corpus composition and filtering outcomes, (2) model optimization, (3) classification model performance, (4) classification ensemble model validation, (5) cross-agency bias mitigation, and (6) real-time monitoring capabilities.

### Document corpus composition and filtering outcomes

From the original 442,437 documents, content filtering reduced the corpus to 99,070 documents based on three criteria: publication between 2019–2025 (July), English language content, and exclusion of categorically unrelated content.

Final classification yielded 2,433 AI-relevant documents from the FDA (3.4 percent), 538 from the EMA (2.4 percent), and 17 from the WHO (0.3 percent) relative to the final time, category, and language filtered dataset, wherever applicable. These proportions illustrate the challenge: for every 100 FDA documents processed, roughly 3 contained AI-relevant regulatory content, while the WHO required processing over 5,000 documents to identify just 17 AI-relevant publications.

The distribution of AI-relevant documents across agencies reflects their distinct regulatory mandates: FDA (2,433 documents, 3.4%) and EMA (538 documents, 2.4%) operate as direct authorities issuing binding guidance, while WHO (17 documents, 0.3%) functions primarily as a global coordination body providing high-level policy frameworks rather than detailed regulatory guidance (World Health Organization, 2025). This disparity illustrates how regulatory fragmentation manifests not only in conflicting approaches but also in the uneven distribution of regulatory activity across institutions with different jurisdictional authorities (see Table 1: Results).

**TABLE 1:** Document Funnel across regulatory agencies.

Agency	Filter Stage	Count	%
FDA	Initial collection	85,759	100.0
	Time & Content filter^a^	71,672	83.6
	AI classification	2,433	3.4
EMA	Initial collection	298,534	100.0
	Language filter^b^	85,978	28.8
	Time & Content filter	22,291	7.5
	AI classification	538	2.4
WHO	Initial collection	58,144	100.0
	Time & Content filter	5,107	8.8
	AI classification	17	0.3
Total	Initial collection	442,437	100.0
	All filters	99,070	22.4
	AI classification	2,988	3.0

^a^ FDA time filter applied but unreliable—timestamps reflect both content and technical updates.

^b^ All EMA documents have English versions. Percentages relative to initial collection per agency. AI classification percentage relative to filtered collection.

### Model optimization

Error correlation analysis revealed that semantic and keyword-based approaches captured complementary information (correlation coefficients 0.41–0.53), supporting the OR-ensemble design. These moderate correlations indicate that classifiers make different types of errors, allowing ensemble combinations to improve performance by reducing the probability that all methods simultaneously misclassify the same document. Grid search optimization identified the optimal OR-ensemble model: (ai_keyword_combined_score ≥0.33, avg_score ≥0.49). These thresholds correspond to extreme tail positions (+2.36σ and +1.87σ from distribution means), indicating that AI-relevant content differs substantially from typical regulatory writing patterns, with explicit AI terminology appearing less frequently than contextual AI concepts. This asymmetry informed the ensemble design, which used complementary detection mechanisms to capture the varied ways regulatory bodies discuss AI technologies.

(See Supplementary Results Section 1; Supplementary Figure S1 for complete error correlation analysis, score distribution figures, and threshold optimization details).

### Classification model performance

The dual-scoring classification model achieved 71.7% Test Recall and 94.6% High Priority Recall, with a 3.0% Positive Rate (Powers, 2011). These performance metrics reflect the fundamental challenge of rare-event detection in large document collections, where achieving high recall without excessive false positives requires careful threshold optimization.

Individual scoring methods demonstrated expected precision-recall tradeoffs, with semantic similarity achieving 94.6% High Priority Recall at 3.7% Positive Rate. The combination of both methods improved robustness by capturing documents through complementary detection mechanisms, addressing the varied linguistic patterns across regulatory agencies. This performance differential reflects the classification challenge: documents with explicit AI terminology are more readily identified by semantic similarity, whereas those discussing AI in broader regulatory contexts require more sophisticated contextual analysis.

(See Supplementary Table S1 for complete individual method performance metrics; Supplementary Table S2 for grid search results across all tested configurations).

### Classification ensemble model validation

Boundary analysis validated ensemble performance on ambiguous classification cases near decision thresholds. The OR-ensemble achieved substantial recall improvements at decision boundaries without proportional increases in false positive rates: at the AI keyword narrow boundary, ensemble recall improved by +50 percentage points (43.8% → 93.8%) with only +13.9 percentage point increase in boundary positive rate (42.7% → 56.6%); at the average reference narrow boundary, ensemble recall improved by +15 percentage points (60.0% → 75.0%) with only +6.0 percentage point increase in boundary positive rate (40.4% → 46.4%). This disproportionate improvement in recall versus positive rate demonstrates genuine discriminative capability rather than indiscriminate positive bias.

The asymmetric synergy pattern reveals why OR-ensemble logic is effective: documents near the AI keyword threshold often use implicit AI references that keyword methods struggle with, but reference similarity captures through semantic context. Conversely, documents near the average reference threshold contain moderate semantic similarity but may lack explicit terminology, where keyword scoring provides complementary signal. This explains the larger ensemble benefit at the AI keyword boundary (+50 pp) versus average reference boundary (+15 pp)—the methods address different failure modes.

(See Supplementary Results Section 2 for complete boundary analysis findings; Supplementary Figure S2 for test dataset ensemble synergy analysis; Supplementary Figure S3 for full corpus boundary analysis).

### Regulatory intelligence analysis

To demonstrate the analytical potential of the document corpus, a preliminary trend analysis was conducted on a representative subset of classified documents. This proof-of-concept illustrates the types of insights that could be systematically generated through the proposed insights framework.

Cross-Agency Thematic Patterns: Exploratory content analysis of document titles and abstracts suggested divergent regulatory priorities. FDA documents more frequently referenced validation terminology (68% vs. 31% for EMA), while EMA documents emphasized governance language (52% vs. 23% for FDA). Studying these patterns could reveal different regulatory mandates and approaches to AI oversight.

Document Type Distribution: The most common document types were regulatory guidance, discussion papers, technical reports, and formal regulations. Guidance documents provided non-binding recommendations for industry implementation. Discussion papers represented preliminary regulatory thinking intended to solicit stakeholder feedback before formal policy development, such as FDA’s “Using Artificial Intelligence & Machine Learning in the Development of Drug & Biological Products” discussion paper (U.S. Food and Drug Administration, 2021). Technical reports documented regulatory science investigations and pilot program results that establish the evidence base for policy decisions, including FDA’s evaluation frameworks for AI-enabled medical devices and WHO’s assessments of AI implementation challenges in healthcare systems. Regulatory documents often emphasized regulatory evolution, particularly in adapting frameworks for data-intensive AI tools in clinical development and established legally enforceable requirements.

### Cross-agency bias mitigation

Initial classification using FDA-only reference documents (n = 27) revealed systematic bias across agencies. The optimized classifier achieved 2.8% positive classification rate on FDA documents but only 1.6% on EMA documents, suggesting under-detection of AI-relevant content in non-U.S. regulatory frameworks.

To address this bias, the reference document set was expanded to include 8 EMA examples (final n = 35), incorporating documents that used European regulatory terminology and policy frameworks, and keyword taxonomies were enhanced with jurisdiction-specific terminology. Post-mitigation validation demonstrated bias reduction: positive classification rates converged to 3.4% (FDA) and 2.4% (EMA), indicating improved cross-jurisdictional generalizability.

The substantial improvement in EMA classification rates (1.6% → 2.4%) demonstrates the effectiveness of diversified reference document selection for cross-agency generalization. This 50% increase in EMA document detection indicates that the initial FDA-centric model was systematically missing EMA documents that discussed AI concepts using European regulatory language, policy frameworks, or document structures not represented in the original reference set.

### Real-time monitoring capabilities

The system maintains real-time regulatory intelligence through automated weekly pipelines that scrape agency sitemaps, download new content, generate embeddings, and update classification scores. Each agency update cycle completes in under 1 h when run weekly, with the option to trigger the pipelines on demand as needed.

New documents are identified by comparing current sitemap contents against existing records. Between January and April 2025, automated monitoring identified 107 new AI-relevant documents from 5,869 updated FDA documents (1.8% Positive Rate), and 42 from 1,219 updated documents from May-July 2025 (3.4% Positive Rate). These classification rates align with our initial corpus analysis (3.0% Positive Rate), suggesting stable classifier performance on new documents. While we cannot estimate precision without manual validation of these newly classified documents, the consistency in classification rates demonstrates the framework’s capacity for ongoing regulatory monitoring without manual intervention.

## Discussion

The study demonstrates that AI-powered approaches can effectively federate AI-related drug regulations across jurisdictions through three methodological innovations. First, automated data pipelines integrate directly with regulatory sources (FDA, EMA, WHO), enabling continuous monitoring without manual tracking limitations that create information delays. This represents a significant advancement over static, point-in-time analyses that characterize existing regulatory intelligence efforts.

Second, a domain-specific semantic similarity methodology was developed to replace traditional keyword-matching approaches. The framework uses semantic similarity scoring combined with contextual concept analysis to identify relevant regulatory content, even when documents use diverse terminologies or regulatory language across agencies. The optimized semantic similarity scoring achieves 72% overall recall and 95% recall for highly relevant content, demonstrating the effectiveness of this approach in specialized regulatory domains.

Third, the system’s extensible architecture supports straightforward updates to reference documents and keyword taxonomies, enabling adaptation to emerging regulatory themes without complete system redesign. As regulatory language evolves, new exemplar documents can be added to the reference set and domain-specific terminology can be incorporated into keyword taxonomies through manual curation, maintaining classification performance without retraining.

Several important findings emerged from the analysis. The diversity of AI-related regulatory documents exceeded initial expectations, with valuable insights captured not only in formal guidance documents but also in meeting minutes, advisory committee presentations, and third-party research papers. This finding highlights the importance of comprehensive data collection strategies that extend beyond traditional regulatory publication channels.

Initial disparities in document yield between agencies were traced to classification model bias rather than true regulatory output differences. These disparities were successfully corrected by diversifying reference documents and expanding keyword taxonomies to include jurisdiction-specific terminology, resulting in converged classification rates of 3.4% (FDA) and 2.4% (EMA) following bias mitigation.

### Validated system capabilities and current limitations

The current implementation of AICURIS has been validated as a regulatory intelligence system for the identification and classification of AI-relevant regulatory documents using a fixed reference set and predefined keyword taxonomies. Validation has been conducted exclusively on English-language regulatory documents issued by the FDA, EMA, and WHO. Within this scope, the system reliably supports descriptive mapping and structured comparison of AI-related regulatory content across these sentinel authorities.

However, using a fixed reference set constrains the system’s ability to capture newly emerging regulatory concepts as AI-related terminology evolves. In addition, validation is limited to English-language documents and to regulatory systems rooted largely in Western legal and scientific traditions. As a result, the current system should not be interpreted as providing comprehensive global regulatory coverage or as capturing the full diversity of regulatory philosophies, terminologies, or document structures used across jurisdictions.

### Linguistic, cultural, and jurisdictional constraints

The English-only validation introduces two distinct limitations. First, embedding model performance and classification accuracy for non-English regulatory texts remain unvalidated. Second, and more fundamentally, the regulatory authorities included to date reflect closely related regulatory cultures. The FDA and EMA operate within comparable legal and scientific frameworks, whereas the WHO primarily serves as an international coordinating body rather than a direct regulatory authority.

Consequently, claims regarding global regulatory harmonization should be interpreted as exploratory and inferential. Extending AICURIS to truly global regulatory intelligence will require systematic validation across regulatory systems with differing legal traditions, regulatory philosophies, and linguistic conventions, including agencies such as the PMDA, MHRA, and NMPA, as well as regulators in low- and middle-income countries. Without such validation, global applicability cannot be assumed.

### Scope and generalizability constraints

The proof-of-concept analyses (cross-agency thematic patterns, document type distributions) represent preliminary explorations of document subsets to demonstrate analytical potential rather than comprehensive regulatory intelligence. These analyses have not been systematically validated and should be interpreted as illustrative examples rather than definitive findings. Additionally, while this study focused exclusively on AI-related pharmaceutical regulations, the methodological framework could potentially extend to other regulatory intelligence domains (e.g., real-world evidence, pharmacogenomics, pharmacovigilance) with appropriate adaptations, including domain-specific reference document curation, keyword taxonomy development, validation dataset creation, and threshold optimization. However, expansion faces barriers, including domain-specific variations in regulatory language, limited availability of high-quality reference documents for emerging topics, and computational costs associated with processing large document corpora.

### Future development roadmap

Future development will focus on three key areas: (1) adaptive classification using automated concept discovery to identify emerging regulatory terminology, (2) multilingual coverage expansion to major pharmaceutical markets, and (3) automated insights generation through validated analytical frameworks, including topic modeling, cross-jurisdictional comparisons, and temporal trend analysis. All extensions will require rigorous validation before deployment in regulatory contexts and are not intended to replace expert regulatory judgment. Any future analytical capabilities will remain subject to human oversight and expert validation, ensuring appropriate use in regulatory decision-making contexts.

(See Supplementary Discussion for detailed development roadmap including adaptive classification methods, multilingual expansion plans, automated insights generation approaches, and governance frameworks).

## Conclusion

As the AI-enabled ecosystem for human therapeutics continues to evolve, relevant global drug regulations are being written. The need for accessible, harmonized regulatory frameworks has never been more urgent. In this study, we introduce the first AI-powered, continuously updated system capable of monitoring and classifying AI-related regulatory content across major global agencies. The six-part analysis demonstrates the system’s ability to identify relevant documents with high recall, mitigate jurisdictional bias, and enable ongoing automated tracking with frequent update cycles. The sharp rise in AI-related regulatory publications alongside varying regional emphases highlights the growing complexity and urgency of aligning standards across borders.

Looking ahead, the framework provides a scalable foundation for building global convergence in AI-related regulations. Future iterations could integrate additional regulatory bodies and multilingual documents, expand to include regulatory documents for medical devices, and support automated policy benchmarking.

We envision AICURIS as a catalyst for international collaboration between drug developers and regulators, particularly benefiting low- and middle-income countries by democratizing access to cutting-edge regulatory intelligence. Ultimately, this approach can foster a more inclusive, transparent, and harmonized global regulatory environment that accelerates the safe and equitable deployment of AI-enabled human therapeutics worldwide.

## Data Availability

The raw data supporting the conclusions of this article will be made available by the authors, without undue reservation.
