# Supplementary material for: Federating AI-related regulations for human therapeutics: an AI-enabled, continuously updating regulatory intelligence system
Source: Front Drug Saf Regul. 2026 Mar 9;6:1718379. doi: 10.3389/fdsfr.2026.1718379 (PMC13006491; doi:10.3389/fdsfr.2026.1718379)
Supplement: Supplementary file 1 [file Supplementaryfile1.pdf]

SUPPLEMENTARY MATERIALS

Federating AI-Related Regulations for Human Therapeutics: An AI-Enabled, Continuously Updating Regulatory Intelligence System

TABLE OF CONTENTS

SUPPLEMENTARY METHODS

SUPPLEMENTARY RESULTS

SUPPLEMENTARY DISCUSSION

SUPPLEMENTARY DISCLOSURES

==

SUPPLEMENTARY METHODS

Section 1: Data Acquisition Technical Implementation

Detailed Technical Tools and Protocols

A systematic data collection framework was applied using Python-based tools: BeautifulSoup (Richardson, 2025) for parsing HTML content from regulatory websites and PyMuPDF (Artifex Software, n.d.) for extracting text from PDF documents, which comprise the majority of regulatory publications. Structured sitemap traversal and content extraction protocols ensured comprehensive coverage. Metadata, including publication date, agency, document type, and language, was preserved to enable temporal trend analysis and cross-jurisdictional comparative studies. The architecture supported continuous ingestion from agency websites and RSS feeds.

Framework Architecture and Computational Efficiency

The framework architecture prioritizes computational efficiency and scalability, enabling deployment across multiple regulatory agencies with minimal resource overhead. Rather than requiring agency-specific systems, the unified pipeline processes diverse document formats and regulatory languages through standardized extraction protocols, reducing both the development costs of building separate data processing systems for each agency and ongoing maintenance requirements.

FDA Time Filtering Limitation

FDA sitemap metadata contains 'last-modified' timestamps that do not reliably distinguish between content updates and technical website modifications. A timestamp from 2024 may reflect either substantive content revision or routine technical

maintenance of a document originally published in 2015. The 2019+ time filter was applied to FDA documents, but this approach may have inadvertently included irrelevant content (technical updates to old documents). Manual inspection of random samples (n=100) from the filtered set confirmed predominant coverage of the target timeframe (2019-2025), but the reliability of FDA temporal filtering remains lower than for EMA and WHO, where timestamp metadata more accurately reflects content publication dates.

Section 2: Reference Document Set Development

Detailed Selection Process

Two authors (RS, ES) with expertise in regulatory science and AI applications independently reviewed publicly available AI-related regulatory guidance published by FDA, EMA, and WHO through mid-2024. Documents were nominated for inclusion based on three criteria: (1) explicit mention of AI/ML technologies in regulatory contexts, (2) representation across different document types (guidance documents, discussion papers, technical reports), and (3) coverage of diverse AI applications spanning drug development, manufacturing, and drug safety surveillance. The team met to achieve consensus on the final 34 documents, ensuring balanced representation across agencies (FDA: 27, EMA: 7) and document types.

Section 3: Synthetic Reference Document Construction

Construction Methodology

A synthetic "ideal" document was constructed by combining comprehensive keywords and regulatory concepts from three domains: AI terminology (67 terms including machine learning, neural networks, generative AI, explainable AI), legal/regulatory language (34 terms including validation, compliance, risk assessment, governance), and biomedical terminology (71 terms including clinical trials, pharmacovigilance, precision medicine).

Rationale and Validation

This represents a generalized concept space and serves as a controlled semantic construct designed to anchor similarity scoring across heterogeneous regulatory language. This approach was motivated by the observation of how AI regulatory concepts are referenced across regulatory websites and context types (e.g., technical language). This reduces reliance on exact terminology overlap and improves recall for relevant documents that referenced AI, regulatory and biomedical terminology implicitly or using non-standard phrasing.

Validation of the document was conceptual rather than empirical: its content was reviewed to ensure broad coverage of recurring regulatory themes while avoiding

verbatim overlap with any single source document. We acknowledge that this abstraction may introduce conceptual bias by the keyword taxonomy used, potentially under-representing emerging or context-specific regulatory framings.

Section 4: Test Document Set Development

Independent Sampling Strategy

To validate classifier performance without introducing bias from the classification framework, a test set was created through independent keyword-based sampling. A separate set of AI-related keywords was developed (distinct from those used in the classification model) and used the FDA website's native search functionality to identify potentially relevant documents. This keyword-based search included terms such as "artificial intelligence", "machine learning", "algorithm", "neural network", "deep learning", and "predictive model", combined with regulatory context terms like "guidance", "regulatory", "approval", and "validation". This automated sampling process identified 842 documents from the FDA corpus for manual review.

Two-Stage Manual Review Process

Two team members (RS, ES) independently reviewed all 842 documents through a two-stage process to create the test set.

Stage 1 - Relevance Screening: Reviewers assessed the title, abstract, and metadata to identify documents discussing AI/ML/data science technologies within regulatory contexts. Each reviewer classified documents as "relevant" or "not relevant" with disagreements resolved through discussions. This identified 317 AI-relevant documents.

Stage 2 - High-Confidence Classification: For the 317 AI-relevant documents, both reviewers conducted full-text reviews to distinguish documents containing explicit AI regulatory guidance (validation standards, algorithmic transparency requirements, dedicated AI frameworks). This classified 73 documents as "high-confidence."

Final Test Set: After removing contextual duplicates (multiple versions of the same guidance or event), the final test set comprised 127 AI-relevant documents: 38 high-confidence documents (e.g., FDA's "Considerations for the Use of Artificial Intelligence To Support Regulatory Decision-Making") and 89 relevant documents with less explicit AI content (e.g., digital health frameworks mentioning AI capabilities without detailed technical requirements).

Test Set Limitations

The test set was constructed exclusively from FDA documents for three pragmatic reasons: (1) FDA represented the largest document corpus (~85,000 documents),

providing sufficient sampling depth; (2) FDA's website search functionality enabled systematic keyword-based sampling independent of the classification framework; (3) This approach enabled efficient validation resource allocation while establishing baseline classifier performance. The potential FDA-centric bias was subsequently addressed through cross-agency validation (see Cross-Agency Bias Mitigation in Results).

Independence from Classification Framework

The keyword-based sampling approach used to create the test set was intentionally independent from the semantic similarity and keyword scoring classification model. This independence ensures that test set performance metrics reflect genuine classifier validation rather than overfitting to their own feature engineering choices. The keywords used for test set sampling were broader and more generic than the classification model's refined taxonomy, and documents were selected via FDA's search engine rather than the embedding-based similarity scoring.

Important Limitation - Precision Estimation

The test set consists only of documents pre-identified as AI-relevant through manual screening. Therefore, the classifier precision (true negative rate) or false positive rates cannot be estimated, as the test set does not include a representative sample of true negative documents. This design choice reflects the methodological priority: validating the system's ability to capture known AI-relevant content (recall optimization) over estimating false discovery rates.

Estimating precision would require manual review of a random sample of the ~96,000 documents (97% of corpus) classified as non-relevant, which was not feasible given resource constraints. The 3.0% positive classification rate (2,988 documents) represents a manageable manual review burden for regulatory intelligence applications, justifying the recall-prioritized approach.

Section 5: Classification Model Technical Specifications

Semantic Similarity Scoring Technical Details

Vector embeddings (OpenAI, n.d.-a) were generated using OpenAI's text-embedding-3-large model (OpenAI, n.d.-b) from the first 4,096 characters of each document. This length was chosen for two reasons: (1) computational efficiency when processing 400,000+ documents, as full-document embeddings would require substantially greater resources, and (2) regulatory documents typically establish their purpose, scope, and key concepts in opening sections, title, abstract, and initial paragraphs, making early content highly informative for classification.

Cosine similarity scores were calculated against all the 35 (34 actual + 1 synthetic) reference documents, generating the mean cosine similarity (avg_score) between a document and all 35 reference documents and maximum cosine similarity (max_score) with the most similar reference document to each document (Mikolov et al., 2013).

Keyword Similarity Scoring Technical Details

Comprehensive taxonomies were developed covering three critical domains: AI terminology (including machine learning, neural networks, generative AI, and explainable AI concepts), legal and regulatory language (validation, compliance, risk assessment, and governance frameworks), and biomedical terminology (clinical trials, pharmacovigilance, drug development, and precision medicine). The keyword taxonomies were iteratively developed through expert review and expanded to capture emerging AI regulatory terminology. Each document was scored for cosine similarity to domain-specific keyword embeddings comprising 36 AI terms, 41 legal/regulatory terms, and 58 biomedical terms, enabling detection of AI-relevant content even when semantic similarity to reference documents was low.

Seven Scoring Configurations

Seven scoring configurations were systematically evaluated. These seven methods comprise:

(1) avg_score - mean cosine similarity across all 35 reference documents

(2) max_score - maximum cosine similarity to any single reference document

(3) ideal_document_score - similarity to the synthetic ideal document

(4) ai_keyword_combined_score - similarity to a single embedding generated from concatenated text of all 36 AI keywords (e.g., "AI and related concepts including: machine learning, neural networks, generative AI...")

(5) legal_keyword_combined_score - similarity to an aggregate embedding of all 41 legal/regulatory terms

(6) biomedical_keyword_combined_score - similarity to an aggregate embedding of all 58 biomedical terms

(7) all_keyword_combined_score - similarity to an aggregate embedding combining all three keyword domains

The "combined" keyword scores represent conceptual proximity to entire terminological domains rather than matching individual keywords, enabling detection of documents using AI-relevant language even when specific reference document similarity is low.

Individual and OR-Ensemble Classification Models

Individual Classification Models: Each of the seven scoring methods can function as a standalone binary classifier by setting a decision threshold between 0 and 1; documents scoring above the threshold are classified as AI-relevant, those below as not relevant.

OR-Ensemble Scoring Models: OR-ensemble classifiers combine multiple scoring methods using disjunctive logic: a document is classified as AI-relevant if it exceeds the threshold in any of the constituent methods. For example, an (avg_score OR ai_keyword_combined_score) model with thresholds (0.50, 0.33) classifies a document as relevant if either avg_score ≥ 0.50 or ai_keyword_combined_score ≥ 0.33.

Section 6: Performance Metrics Mathematical Formulations

Test Recall = TP_test / (TP_test + FN_test)

where TP_test = true positives in the 127-document test set, and FN_test = false negatives in the test set. Test Recall therefore measures the proportion of known AI-relevant documents in the curated test set that were correctly identified by the classifier.

High Priority Recall = TP_high / (TP_high + FN_high)

where TP_high = true positives in 38 high-confidence subset, and FN_high = false negatives in high-confidence subset. This metric assesses performance on unambiguous AI regulatory guidance documents.

Under the assumption that the curated test set is representative of canonical AI-related regulatory language, Test Recall and High Priority Recall offer an indicative - but not unbiased - estimate of recall for similar documents in the full corpus.

Positive Rate = Classified_positive / Total_corpus

where Classified_positive = documents exceeding classification threshold(s), and Total_corpus = 99,070 filtered documents. Positive Rate indicates the proportion of the entire corpus classified as AI-relevant, which directly determines manual review burden.

Section 7: Threshold Optimization Methodology

Grid Search Procedure

For each scoring method, a grid search was performed over threshold values [0.0, 1.0] in 0.01 increments. At each threshold value, a Test Recall was computed on the 127-document test set and Positive Rate across the full 99,070-document corpus. The "best" threshold for each individual method was defined as the value achieving ≥70% Test Recall while minimizing Positive Rate (Bergstra & Bengio, 2012).

For the OR-ensemble, a two-dimensional grid search was performed, to identify the model and threshold pair that maximized Test Recall while maintaining acceptable Positive Rate (<5%).

Recall Prioritization Strategy

Pharmaceutical development decisions carry high costs for missed regulatory guidance (false negatives), while false positives impose manageable manual review burden. Missing critical AI regulatory requirements could delay drug development or cause non-compliance, whereas reviewing ~3,000 classified documents (as per the 3% observed Positive Rate) remains feasible for regulatory intelligence teams. Therefore, the optimization prioritized recall (capturing known AI-relevant documents) over precision (minimizing false positives), accepting the low-prevalence classification challenge inherent to rare-event detection in large document collections.

The OR logic directly supports the recall-maximization strategy: documents need only satisfy one detection pathway rather than both, increasing the probability of capturing AI-relevant content expressed through either explicit terminology or contextual similarity to known AI regulatory documents (Wang & Yao, 2012).

Section 8: Ensemble Model Boundary Analysis Methodology

Boundary Region Definitions

For each scoring method (AI keyword score and average reference score), two boundary regions were defined:

- Narrow boundary: Documents within ±0.01 of the threshold value

- Wide boundary: Documents within ±0.5σ of the threshold value (where σ is the standard deviation of that score's distribution)

For example, with an AI keyword score threshold of 0.33 and σ=0.053, the narrow boundary includes documents with scores [0.32, 0.34], while the wide boundary includes documents with scores approximately [0.30, 0.36].

Metrics Computation

For documents within each boundary region:

Boundary Positive Rate = N_positive_b / N_total_b

where N_positive_b = number of documents in the boundary region classified as positive (exceeding threshold), N_total_b = total number of documents in the boundary region

Individual Boundary Recall = TP_individual_b / (TP_individual_b + FN_individual_b)

where TP_individual_b = true positives out of all test documents in the boundary region when using only the individual scoring method, FN_individual_b = false negatives out of all documents in the boundary region when using only the individual scoring method

OR-Ensemble Boundary Recall = TP_ensemble_b / (TP_ensemble_b + FN_ensemble_b)

where TP_ensemble_b = true positives out of all test documents in the boundary region when using OR ensemble (either score exceeds threshold), FN_ensemble = false negatives out of all test documents in the boundary region when using OR ensemble

Ensemble Improvement = OR-Ensemble Boundary Recall - Individual Boundary Recall

This metric quantifies the recall gain achieved by combining both scoring methods versus using each method alone. Positive values indicate synergistic benefit from the ensemble approach.

This analysis was applied to both the 127-document test set and the full 99,070 document corpus to assess ensemble performance across different scales and document contexts.

Section 9: Technical Implementation and Reproducibility

Technology Stack

The technical stack included Python for scraping, BeautifulSoup for HTML parsing, PyMuPDF for PDF content extraction, and OpenAI's embedding model for semantic analysis. Data pipelines were developed for each agency by traversing the website sitemaps.

Cloud Infrastructure

Cloud-based infrastructure (Amazon S3) supported documents and Amazon Web Services Relational Database Service (AWS RDS) was used for embedding vector storage and maintaining logs for extraction activities (Amazon Web Services, Inc., n.d.-a).

Reproduction Parameters

Reproduction requires constructing a diversified reference document set of ~25 or more examples, building comprehensive keyword taxonomies, and validating thresholds using test sets of 100–200 documents. These parameters reflect the practical constraints and

empirical findings from the development process: it was found that ~25 reference documents provided adequate coverage of regulatory document diversity while remaining manageable for manual curation, and 100-200 test documents offered sufficient validation samples while balancing annotation effort with statistical reliability for threshold optimization. This enables independent verification of classification performance across different regulatory contexts.

SUPPLEMENTARY RESULTS

Section 1: Model Optimization and Error Correlation Analysis

Error Correlation Between Scoring Methods

Error correlation analysis between classification mistakes made by different scoring methods revealed that semantic and keyword-based approaches had moderate correlation coefficients ranging from 0.41 to 0.53, indicating complementary rather than redundant information capture. These moderate correlations are well below the 0.7 threshold where ensemble approaches provide theoretical performance benefits. When classifiers make uncorrelated errors, combining their predictions can improve overall performance by reducing the probability that all methods simultaneously misclassify the same document (Hastie et al., 2009). Artifex Software. (n.d.). PyMuPDF documentation. Retrieved August 18, 2025, from https://pymupdf.readthedocs.io/

Grid Search Optimization Results

Grid search optimization identified the optimal OR-ensemble model: (ai_keyword_combined_score ≥ 0.33, avg_score ≥ 0.49). These thresholds correspond to +2.36σ and +1.87σ from their respective distribution means, ensuring high specificity while maintaining recall through complementary detection mechanisms.

Threshold Position Interpretation

The extreme tail positioning indicates that AI-relevant content differs substantially from typical regulatory writing patterns, with explicit AI terminology (2.36σ threshold) appearing less frequently than contextual AI concepts (1.87σ threshold). This asymmetry informed the ensemble design - using complementary detection mechanisms to capture the varied ways regulatory bodies discuss AI technologies.

Section 2: Complete Boundary Analysis Findings

Overall Performance (Test Dataset N=127)

Test Recall:

- AI keyword score alone: 50.4%

- OR-Ensemble: 71.7%

- Average reference score alone: 66.1%

This overall view shows the OR-ensemble captures more documents than either individual method, consistent with OR logic combining both pathways.

Narrow Boundary Analysis Results

AI Keyword Narrow Boundary Performance (N=16 test documents within ±0.01 of threshold 0.33):

- AI keyword Individual Boundary Recall: 43.8% (7/16 documents correctly classified)

- OR-Ensemble Boundary Recall: 93.8% (15/16 documents correctly classified)

- Ensemble improvement: +50.0 percentage points

This +50 pp gain demonstrates that documents with borderline AI keyword scores are often captured by strong semantic similarity to reference documents, illustrating complementary detection mechanisms.

Average Reference Narrow Boundary Performance (N=20 test documents within ±0.01 of threshold 0.49):

- Avg Reference Individual Boundary Recall: 60.0% (12/20 documents)

- OR-Ensemble Boundary Recall: 75.0% (15/20 documents)

- Ensemble improvement: +15.0 percentage points

The smaller gain at this boundary reflects that documents with borderline semantic similarity often already contain sufficient AI terminology for keyword-based detection, resulting in less pronounced synergy.

Wide Boundary Analysis Results (±0.5σ)

AI Keyword Wide Boundary (N=52 test documents):

- AI keyword Individual Boundary Recall: 46.2%

- OR-Ensemble Boundary Recall: 73.1%

- Ensemble improvement: +26.9 percentage points

Average Reference Wide Boundary (N=47 test documents):

- Avg Reference Individual Boundary Recall: 63.8%

- OR-Ensemble Boundary Recall: 72.3%

- Ensemble improvement: +8.5 percentage points

Selective Classification Demonstration

The ensemble demonstrates selective classification capability beyond simple positive-bias:

Boundary Recall Ensemble Improvement:

- AI Narrow Boundary: +50.0 pp improvement (43.8% → 93.8%)

- Avg Reference Narrow Boundary: +15.0 pp improvement (60.0% → 75.0%)

Boundary Positive Rate Increase (Full Corpus):

- AI Narrow Boundary (N=806): +13.9 pp (42.7% → 56.6%)

- Avg Reference Narrow Boundary (N=1,977): +6.0 pp (40.4% → 46.4%)

The disproportionate improvement on Boundary Recall (+50 pp) versus Boundary Positive Rate increase (+13.9 pp) at the AI Narrow Boundary demonstrates that the ensemble adds genuine discrimination capability rather than indiscriminate positive bias. A non-selective classifier would increase both metrics proportionally.

Interpretation

The asymmetric synergy pattern reveals why OR-ensemble logic is effective for this classification task. Documents near the AI keyword threshold often use implicit or contextual AI references that the keyword-based method struggles with, but the reference similarity method captures through semantic context. Conversely, documents near the average reference threshold typically contain moderate semantic similarity to AI regulatory documents but may lack explicit AI terminology, cases where keyword scoring provides complementary signal. This explains the larger ensemble benefit at the AI keyword boundary (+50 pp) versus average reference boundary (+15 pp): the methods address different failure modes.

SUPPLEMENTARY DISCUSSION

Future Development Roadmap

Several capabilities described below represent planned extensions rather than current system outputs.

Adaptive Classification Enhancement

Planned work includes implementing automated concept discovery methods using large language models to identify emerging terminology patterns in newly issued regulatory documents and to expand classification criteria accordingly. These capabilities have not yet been implemented or validated.

Multilingual Regulatory Coverage

Expanding coverage to major pharmaceutical markets such as Japan, China, Canada, Brazil, Australia, and regions within the EU will require the development and validation of language-specific classification models trained on multilingual regulatory corpora. This presents both technical and methodological challenges and remains outside the scope of the current system.

Automated Insights Generation

While the current system supports document identification and categorization, it does not generate automated policy interpretations or predictive analyses. Future work may explore layered analytical approaches, including topic modeling, cross-jurisdictional comparisons, and temporal trend analysis, potentially supported by retrieval-augmented generation methods. Any such capabilities would require rigorous validation prior to use in regulatory contexts and are not intended to replace expert regulatory judgment.

Governance, Validation, and Stakeholder Engagement

Given the high-stakes nature of regulatory decision-making, any future analytical or synthesis capabilities must remain subject to human oversight and expert validation. The long-term vision includes the development of structured review and validation processes involving regulators, industry stakeholders, and subject-matter experts. These processes would be designed to ensure analytical accuracy, transparency, and appropriate use, while supporting constructive dialogue on AI governance and regulatory alignment.

SUPPLEMENTARY DISCLOSURES

Disclosures (Financial, CoI, AI-Assistance)

Data Source Ethics: This research exclusively utilized publicly available documents and webpages from agencies' official websites. All data collection activities strictly adhered to each website's terms of use and robots.txt directives. No proprietary, confidential, or access-restricted content was accessed or analyzed. The web scraping methodology implemented respectful request intervals to prevent server overload and ensure compliance with ethical web scraping practices. No attempts were made to circumvent access controls, paywalls, or authentication mechanisms.

Open Access Commitment: All regulatory documents analyzed in this study were published by government agencies and WHO for public access and research purposes. The extracted content, methodologies, and analytical frameworks developed in this research will be made publicly available to support broader regulatory intelligence efforts in the AI and life sciences community.

Generative AI Disclosure: The author(s) verify and take full responsibility for the use of generative AI in the preparation of this manuscript. Generative AI was used. No original content or concepts were generated by generative AI. However, generative AI tools were used to support research, concept validation, and editing. The final content was written by a human author and has been fully reviewed for accuracy and quality.

Tool Accessibility and Costs: This research utilized paid commercial tools including:

OpenAI text-embedding-3-large

Amazon S3 storage

Amazon RDS database

Amazon Elastic Compute (EC2)

Funding for computational infrastructure was provided by Northastern University. No external grants or pharmaceutical industry funding supported this work.
